# Primary and metastatic squamous cell carcinoma of the thyroid gland: Two case reports

**DOI:** 10.1515/biol-2022-0475

**Published:** 2022-09-16

**Authors:** Xing Zhao, Pengyu Hao, Jiangbei Tian, Jirun Sun, Dawei Chen, Zhehui Cui, Libo Xin, Yanmin Song, Gang Zhang

**Affiliations:** Department of Pathology, Affiliated Hospital of Chengde Medical College, Chengde, Hebei 067000, P.R. China; Department of General Surgery IV, Baoding First Hospital, Zhonghua Road, Baoding, Hebei 071000, P.R. China; Key Laboratory of Molecular Pathology and Early Diagnosis of Tumor in Hebei Province, Baoding, Hebei, P.R. China; Department of Pathology, Baoding First Hospital, Baoding, Hebei 071000, P.R. China; Department of General Surgery I, CHENGDE County Traditional Chinese Medical Hospital, Chengde, Hebei 067000, P.R. China; Department of Fever Outpatient, CHENGDE County Traditional Chinese Medical Hospital, Chengde, Hebei 067000, P.R. China; Operation Room, CHENGDE County Traditional Chinese Medical Hospital, Chengde, Hebei 067000, P.R. China

**Keywords:** primary squamous cell carcinoma, metastatic squamous cell carcinoma, thyroid gland, case report

## Abstract

This study reports two cases of squamous cell carcinoma of the thyroid (SCCT) presenting as the thyroid goiter, involving one case of primary squamous cell carcinoma originating from the thyroid (PSCCT) and the other case of secondary SCCT of the thyroid. A retrospective analysis of the clinical and pathological findings was done in this study report. In case 1, the thyroid ultrasound showed multi-hypoechoic well-defined nodules, labeled as 3 using Thyroid Imaging Reporting and Data System, measuring 34.1 mm × 28.9 mm × 30.3 mm and 26.5 mm × 22.2 mm × 23.9 mm in the left in the right lobar thyroid, respectively. The patient underwent surgery and was histologically diagnosed with PSCCT. In case 2, the thyroid ultrasound showed a 25.2 mm × 22.2 mm × 18.8 mm hypoechoic nodule in the right lobar thyroid. The patient underwent a frozen biopsy, the results of which increased suspicion of squamous cell carcinoma. A frozen biopsy was followed by an endoscopic evaluation that detected an ulcerative mass measuring 3.0 cm within the mucosa of esophagus. Due to a scarcity of cases, SCCT is a great challenge for the pathologists and the managing team to come up with the best treatment strategy for the patients.

## 1 Introduction

Squamous cell carcinoma (SCC) is a malignant epithelial tumor that entirely comprises cells with squamous differentiation [1]. The squamous cell carcinoma of thyroid (SCCT) presenting as the thyroid goiter is extremely rare and usually occurs in elderly female patients [2]. The clinical features are similar to other histological types of thyroid carcinoma. However, chronic thyroid diseases, like Hashimoto’s thyroiditis is noted in some cases. Hence, a few of researchers have concluded that the SCCT deriving from metaplasia was often complicated by chronic thyroid diseases [3]. Imaging studies, like esophagoscopy, laryngoscopy, and bronchoscopy help in confirming if the SCC is of primary or secondary origin. The prognosis of primary squamous cell carcinoma originating from the thyroid (PSCCT) is similar to anaplastic thyroid carcinoma. In this study, we have represented two cases of SCCT and analyzed their morphological manifestation and immune phenotype, to further investigate the pathological essential points, the biological behavior, and the diagnosis of the SCCT.

## 2 Case presentation

### 2.1 Case 1

**Figure 1:**
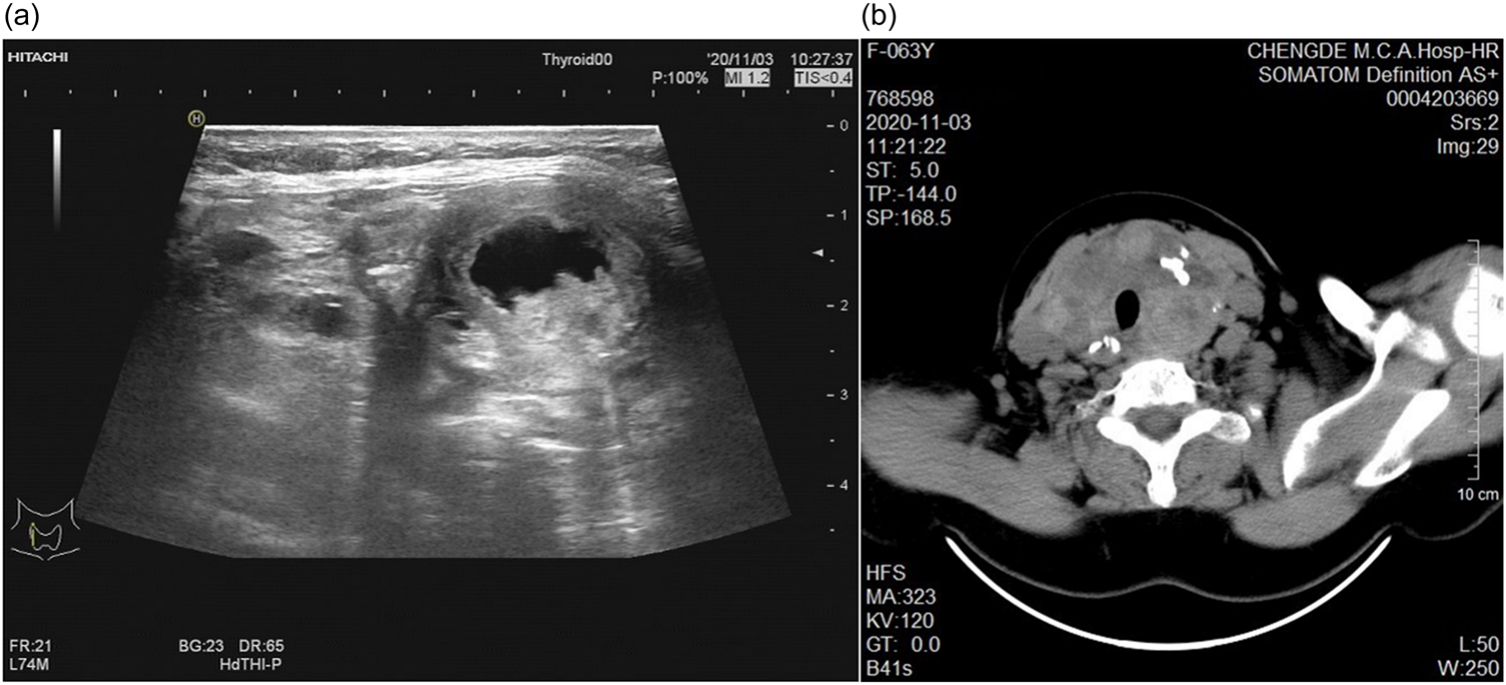
The thyroid ultrasound and CT images of case 1: (a) thyroid ultrasound showed the nodules measuring 34.1 mm × 28.9 mm × 30.3 mm in the left lobar thyroid and 26.5 mm × 22.2 mm × 23.9 mm in the right lobar thyroid and (b) CT showed a multi-low-density image and spot calcification in the whole enlarged thyroid lobe.

A 63-year-old female patient presented with complaints of bilateral neck swelling associated with difficulty in breathing for the past 5 months in 2020. She gave a history of oropharyngeal foreign body sensation, stridor without dysphagia, and hoarseness of voice. She experienced no fever, bodyweight loss, night sweating, malaise, or pain. She also denied a history of neck irradiation or any history of thyroid or other malignancy in her family. Her physical examination revealed that the trachea deviated to the right side. On palpation, a mass approximately measuring 6.0 cm × 5.0 cm on the left side and a mass of 3.0 cm × 2.0 cm on the right side could be felt over the neck. Both the masses were firm with smooth surfaces and moved up and down during deglutition. No palpable cervical lymphadenopathy was present. The rest of the physical examination was found to be normal. The thyroid ultrasound showed multi-hypoechoic well-defined nodules, labeled in Thyroid Imaging Reporting and Data System (TI-RADS) as 3, measuring 34.1 mm × 28.9 mm × 30.3 mm and 26.5 mm × 22.2 mm × 23.9 mm in the left and the right lobar thyroid, respectively. No enlarged lymph nodes in the neck were observed by the ultrasound (Figure 1a). A enhanced computed tomographic (CT) scan revealed a multi-low-density image and spot calcification in the entire thyroid lobe that was enlarged with the compression of the trachea. No other features from the imaging suggested lesions at the other sites of the thyroid lobe (Figure 1b).

**Figure 2:**
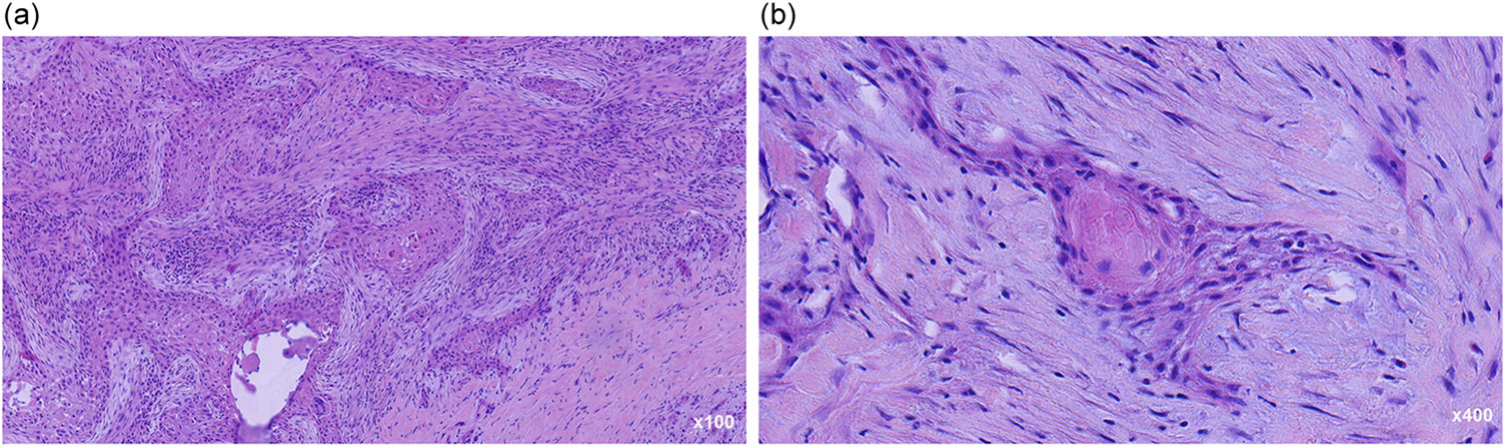
HE images of case 1: (a) these cells were cohesive and arranged in nest and sheet patterns in the low-power field, HE ×100 and (b) presence of keratinization was noted, and the tumor cells exhibited hyper-chromatic nuclei, prominent eosinophilic nucleoli, and abundant eosinophilic cytoplasm in the high-power field, HE ×400.

**Figure 3:**
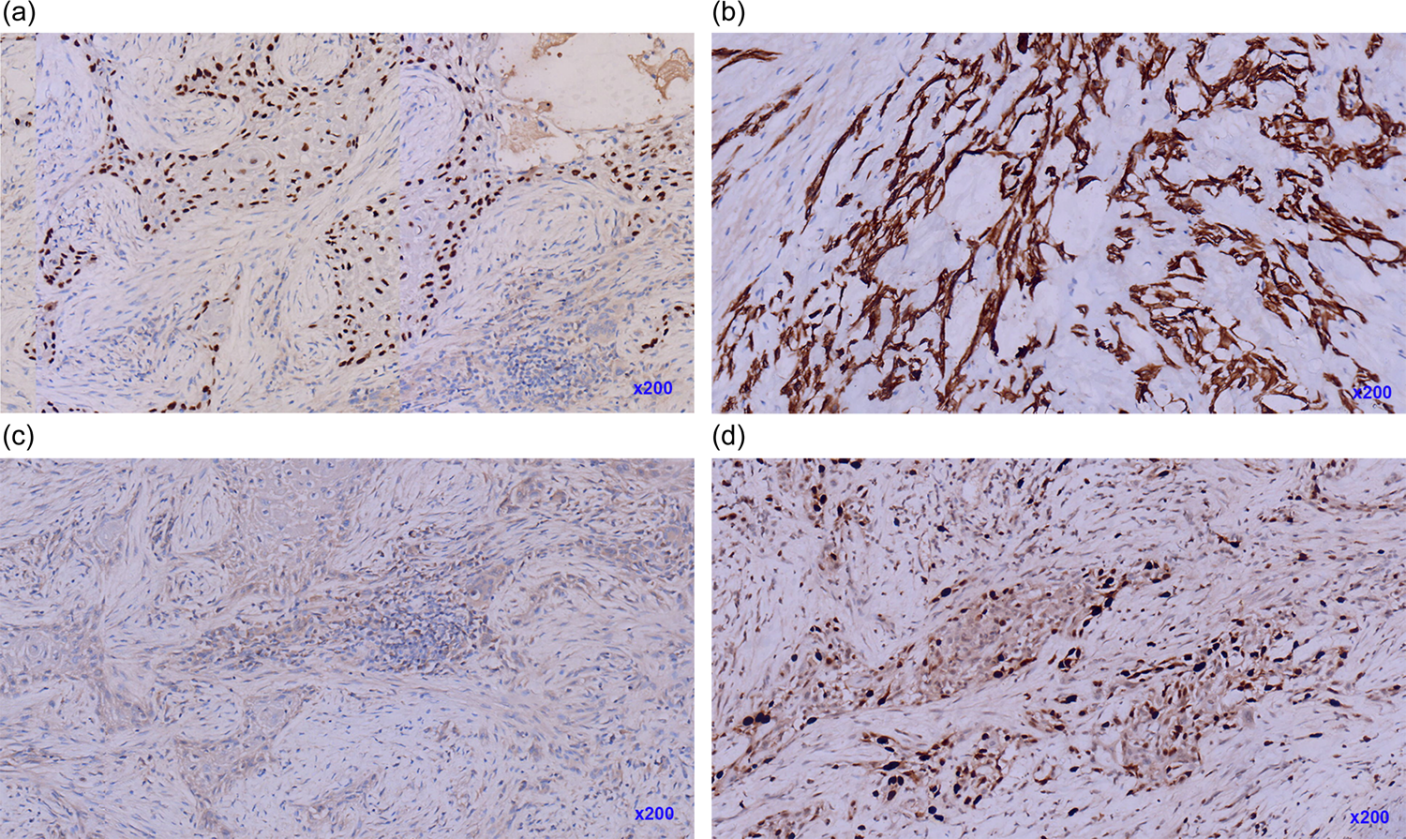
Immunochemistry images of case 1: (a) SCCT cells were positive for p40, p40 immunostaining, ×200, (b) immunochemistry shows SCCT cells positive for CK19, CK19 immunostaining, ×200, (c) SCCT cells were negative for TTF-1, TTF-1 immunostaining, ×200, and (d) Ki-67 proliferative index was approximately 30%, Ki-67 immunostaining, ×200.

The patient underwent a total thyroidectomy. Extensive infiltration of the perithyroidal soft tissue into the tumor was noted during the operation. Grossly, a well-defined mass of 7 cm × 6 cm × 6 cm in the left thyroid gland was found with firm, gray cut surfaces along with a partially calcified cystic lesion. The gross specimen also revealed a 5 cm × 3 cm × 3 cm mass in the right thyroid gland with similar features as were present in the left lesion. The histopathological features were confirmed using hematoxylin & eosin (HE) staining and the stained sections were imaged with an inverted microscope using different magnification times. Microscopically, collagen fibers were seen prominently in the background and the presence of keratinization was noted in the low-power field, which was focally apparent in those with poorly differentiated tumors (Figure 2a). These cells were cohesive and arranged in nest and sheet patterns. The tumor cells exhibited hyper-chromatic nuclei, prominent eosinophilic nucleoli, and abundant eosinophilic cytoplasm. Intercellular bridges and mitotic figures were noted in the high-power field (Figure 2b). On immunohistochemistry (IHC), the tumor cells were positive for CK, P40, CK5/6, and CK19. They were negative for TG, TTF-1, Galectin-3, MC, BRAFV600E, CD117, CD5, and Ki-67. The proliferative index was approximately 30% (Figure 3a–d, Table 1).

**Table 1:** Antibodies and results of immunostaining

Antibody	Clones	Source	Results/Case no.	
			Case 1	Case 2
CK	AE1/AE3	ZSGB	+	+
TG	OT18F2	ZSGB	—	—
TTF-1	8G7G3/1	ZSGB	—	—
P40	BC28	ZSGB	+	+
CK5/6	OTI1C7	ZSGB	+	+
Galectin-3	UMAB157	ZSGB	—	—
CK19	UMAB2	ZSGB	+	+
MC	HBME-1	ZSGB	—	—
Ki-67	EP5	ZSGB	30%	50%
BRAFV600E	RM8	ZSGB	—	—
CD117	EP10	ZSGB	—	/
CD5	EP77	ZSGB	—	/

ZSGB, Zhong Shan Golden Bridge, Beijing.

+: Positive; —: Negative; /: no data.

Based on these results, the patient was diagnosed with primary SCC. The surgery was uneventful, and post-operation the patient had a favorable recovery without complications. During the post-operative period of 7 months, on follow-up, no obvious abnormalities were seen in the patient.

### 2.2 Case 2

A 69-year-old male patient who was admitted to our hospital, presented with a painful cervical mass that developed in the last 15 days, accompanied by dysphagia, and a change in his voice in 2021. The patient had no redness, swelling, cough, bucking, tetany, or hyperhidrosis. He also gave a history of having high blood pressure for the past 8 years but give a negative history of radiation exposure. Physical examination revealed a mass measuring 2.5 cm × 2.0 cm on the right side of the thyroid. The mass was firm with an irregular surface and moved up and down during deglutition.

**Figure 4:**
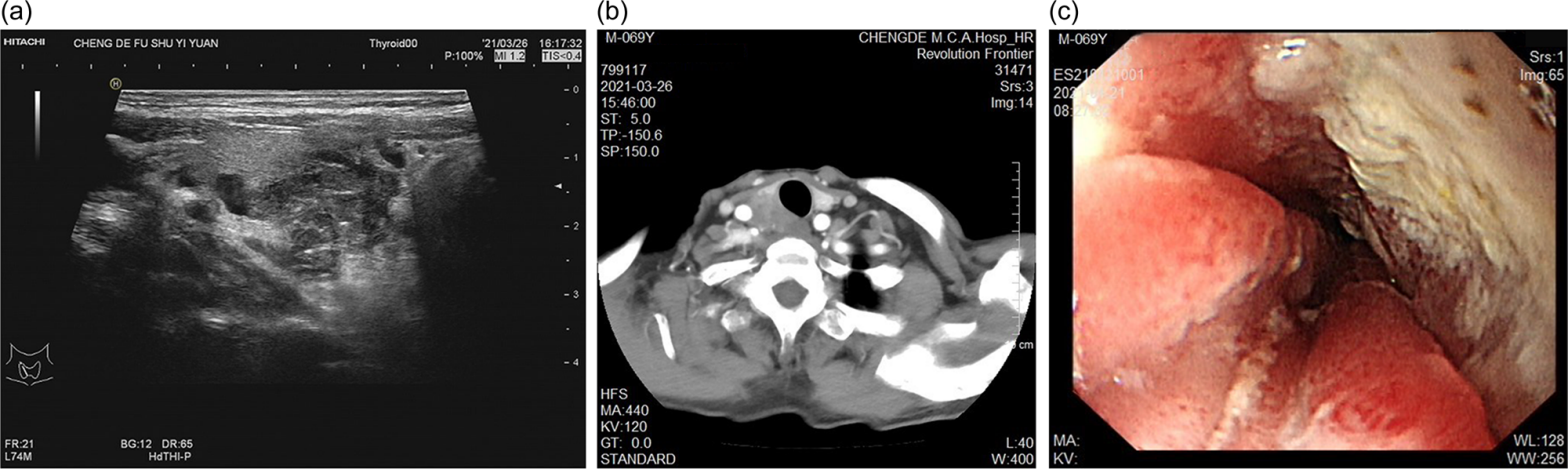
The thyroid ultrasound and CT images of case 2: (a) thyroid ultrasound showed a 25.2 mm × 22.2 mm × 18.8 mm hypoechoic nodule in the right lobar thyroid, (b) CT showed an irregular mass that appeared as soft tissue density in the right lobar thyroid, and (c) upper gastrointestinal endoscopy showed an ulcerative mass measuring 3.0 cm in 28–31 cm from an incisor tooth.

The thyroid ultrasound revealed a 25.2 mm × 22.2 mm × 18.8 mm hypoechoic nodule in the right lobar thyroid labeled as TI-RADS 4b. The left lobar thyroid was unremarkable (Figure 4a). Contrast-enhanced CT revealed an irregular mass that appeared as soft tissue density in the right lobar thyroid. Mass effected the right wall of the trachea, but without invasion of the structures, and there was also no evidence of cervical lymph node metastasis (Figure 4b). During the surgery, firm and wide adhesions were detected between the tumor and the esophagus. The boundary with the carotid artery, bronchial wall, and surrounding tissue was obscure. Hence, only partial resection of the tumor could be done. The patient underwent a frozen biopsy and its result increased suspicion of SCC. Given the findings during the operation, metastasis from the esophagus could not be ruled out. Hence, the frozen biopsy was followed up by an upper gastrointestinal endoscopy that detected an ulcerative mass measuring of 3.0 cm in the esophagus present at the junction between 28 and 31 cm from the incisor tooth (Figure 4c). Another ulcerative tumor located at the gastric fundus was also detected. A pathological examination of an endoscopic biopsy revealed severe atypical hyperplasia of squamous cells both in the upper esophagus and gastric fundus.

**Figure 5:**
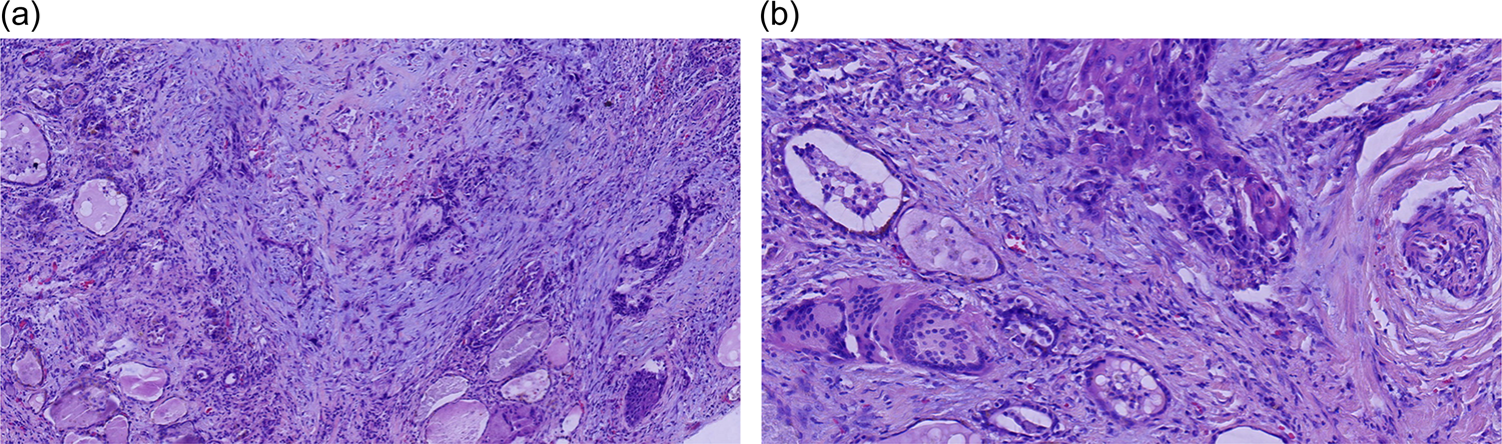
HE images of case 2: (a) solid nests of neoplastic cells are infiltrating widely in the normal thyroid follicles, HE ×100 and (b) tumor cells showed the feature of moderately differentiated SCC, HE ×200.

**Figure 6:**
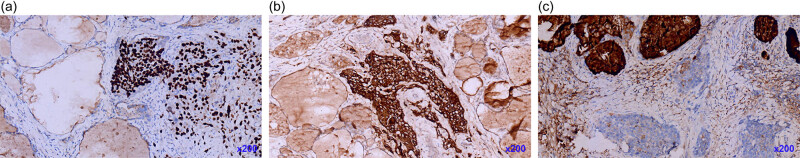
Immunochemistry images of case 2: (a) SCCT cells were positive for p40, p40 immunostaining, ×200, (b) SCCT cells were positive for CK5/6, CK5/6 immunostaining, ×200, and (c) SCCT cells were negative for TG, TG immunostaining, ×200.

HE stain was performed in paraffin-embedded specimens of the thyroid tissue. Solid nests of neoplastic cells widely infiltrating the normal thyroid follicles, showing the features of moderately differentiated SCC were noted (Figure 5a and b). IHC showed tumor cells positive for CK, P40, CK5/6, and CK19, while negative for TG, TTF-1, Galectin-3, MC, BRAFV600E, and Ki-67. The proliferative index was approximately 50% (Figure 6a–c).

Based on these results, the patient was diagnosed with metastatic SCC, from the esophagus. The patient was treated with traditional Chinese medicine and was feeling well 4 months after the diagnosis.


**Informed consent:** Informed consent has been obtained from all individuals included in this study.
**Ethical approval:** The research related to human use has been complied with all the relevant national regulations, institutional policies and in accordance with the tenets of the Helsinki Declaration, and has been approved by the Affiliated Hospital of Chengde Medical College review board.

## 3 Discussion

Thyroid cancer is the most common endocrine tumor, accounting for about 95% of endocrine tumors [4,5]. Some small thyroid nodules, that could not be otherwise noticed in the past, could now be detected due to the improvement of ultrasound. Also, the incidence of thyroid cancer has been steadily rising. Most thyroid cancers originated from the follicular cells. Papillary thyroid carcinoma is the commonly seen histologic type of thyroid cancer that has a good prognosis. SCCT is a rare malignant tumor representing only about 1% of all thyroid cancers. Till now, only hundreds of PSCCT cases have been reported in the literature. Secondary SCCT of the thyroid (SSCCT) is a type of metastatic malignancy from the esophagus, larynx, hypopharynx, lung/trachea, and so on. A recent retrospective study with a larger cohort of PSCCT pointed that there were several different characteristics between PSCCT and SSCCT and with different overall survival time [5]. Thus, it is crucial to distinguish PSCCT and SSCCT for accurate personalized treatment.

The clinical features of the SCCT are unclear due to the absence of the reported cases. SCCT is more common in older women, with a female-to-male ratio of 2:1 [6,7]. Most of the SCCT patients were approximately 50–60 years old. The clinical features of SCCT are similar to other malignant thyroid tumors, presenting as rapidly enlarged masses of the neck with pressure symptoms. In some patients with SCCT, other thyroid diseases are noted, like Hashimoto thyroiditis. Rarely, SCCT is associated with paraneoplastic syndrome. Metastatic thyroid tumors are rare, due to the rapid flow of arterial blood that prevents the adhesion of tumor cells, and the presence of iodine in the thyroid gland that inhibits the proliferation of the tumor cells [8,9]. The metastatic thyroid tumor comprises 2–3% of all thyroid tumors, which could be originating from renal cell cancer, breast cancer, and lung cancer [9,10]. On the basis of the information we have, it can be inferred that SSCCT originating from the esophagus is extremely rare.

The macroscopic appearance of SCCT was typically large involving the entire thyroid with a firm and grayish-white colored surface. On microscopy, the tumor cells revealed squamous differentiation. In some cases of anaplastic and papillary carcinoma, the area of SCC could be detected, but in SCCT other histologic types are forbidden. Extensive infiltration of perithyroidal soft tissue, vessel, and peripheral nerves was often detected.

Unique microscopic morphology and IHC (positive for P40, CK5/6, and negative for TTF-1 and thyroglobulin) are helpful for making the final diagnosis of SCCT. The differential diagnoses include mucoepidermoid thyroid carcinoma, differentiation in papillary carcinoma, squamous metaplasia associated with nodular goiter, and lymphocytic thyroiditis. SSCCT is more common than PSCCT, either due to direct invasion or metastasis. As PSCCT is rare, thyroid metastases should be ruled out before confirming the diagnosis. Imaging studies like esophagoscopy, bronchoscopy, laryngoscopy, and chest X-rays are important for distinguishing PSCCT from SSCCT because these tumors often metastasize to the thyroid. Besides routine examination, immunohistochemical evaluation has a reference value for the diagnosis of PSCCT. The tumor cells of PSCCT are positive for PAX-8 (paired box gene 8), and show P53 over-expression. PAX-8 immunostaining is a novel technique that can help with the diagnostic challenge of distinguishing between PSCCT and SCC metastatic to the thyroid from extrathyroidal sites [11]. Combining PAX-8 immunostaining and clinical diagnostic technique would be useful for accurate diagnosis of the PSCCT. In both the patients that were included in our present study, we excluded the possibility of performing CT in cases 1 and 2. Endoscopic examination and biopsy could confirm the diagnosis of SCCT. The negative immunoreactivity for CD5 and CD117 exclude the SCC of the thymus. The molecular biology of SCCT is unclear. In 50% of cases, abnormal expression of P53 was noted, and in a few cases the loss of P21 expression was noted.

Squamous cell is normally absent in the thyroid, so the tumor origin is confused. There were three hypotheses about the etiology of the SCCT [2,12,13]: (1) in the embryonic origin, SCCT was deduced to originate from the remnant branchial arch or the thyroglossal duct, (2) chronic thyroid diseases are noted in some cases, hence, a few researchers deduced that SCCT is derived from metaplasia complicated with chronic thyroid diseases, and (3) de-differentiation of existing anaplastic, papillary, or medullary carcinomas.

In PSCCT cases, complete surgical resection followed by postoperative radiotherapy is the first choice of treatment whenever possible. The patients should then be closely monitored after surgery to detect recurrence as soon as possible. Since, sometimes a complete surgical resection is not possible. This makes tumor management difficult. In such cases, radiotherapy is the main treatment choice because such tumors usually do not respond to chemotherapy [14–16].

Due to the extensively diffuse infiltration of PSCCT to the trachea, esophagus, and major blood vessels, the survival rate of patients is less than a year after the diagnosis, similar to anaplastic thyroid carcinoma. Lymphadenectomy and extended resections have no impact on patient survival [17].

## 4 Conclusion

Both PSCCT and SSCCT are diagnostic challenges for us. Pathologists should keep in mind SCCT, especially in thyroid frozen biopsy, and remind the surgeons to perform further examinations to distinguish PSCCT from SSCCT because histology has limitations in this tumor and SSCCT is a diagnosis of exclusion. Early and accurate diagnosis of PSCCT or SSCCT is critical for designing reasonable treatment and improving survival rate.
